# Editors and Editing

**Published:** 1886-02

**Authors:** Garrett Newkirk

**Affiliations:** Chicago, Ill.


### EDITORS AND EDITING.

BY GARRETT NEWKIRK, M. D., CHICAGO, ILL.

Dr. Cushing is about right.* We do not have enough editing. Not long since the *New York Tribune* made some keen criticisms upon the Chicago papers, the big "blanket sheets," to this effect: that in the truest sense they were not edited; that the business of an editor was to cull, sift, cut down, change, and make presentable the materials offered. The "blanket" sheets, it was said, were simply masses, gathered into an indiscriminate pile, slung together, etc. The editor shirked his responsibilities, and forced the reader to do the sifting at a great waste of time, and often to his disgust. I do not know of any dental or medical journal so poorly edited as are some of our dailies. There is none better than the INDEPENDENT PRACTITIONER, yet there is still an application in the *Tribune's* remarks.

It is the editor's business to *edit.* According to Webster, the editor not only collects matter, but he is "*especially one who prepares, superintends, revises, and corrects a book, magazine, newspaper, etc., for publication.*" Something may be offered which demands publication, but which is manifestly wrong in part, with perhaps mischievous tendencies. If the editor thinks the world would be no better off for its publication, he should cast it out. If he thinks the world would be benefited by revision, correction or elimination, he should insist upon exercising his prerogative. A part of the subscription price paid the journal is for *editing;* the subscribers pays for having matter presented in the best shape possible, according to the editor's judgment. The feelings of the author of an article have nothing to do with the affair. It is the value of the matter itself that is to be considered. When questionable doctrines are published as parts of papers, otherwise good, the editor should, as Dr. Cushing suggests, make some editorial comment, or see to it that some one else makes a fair criticism, instead of letting such matter go forth, as it often does, unquestioned.

---

* See the INDEPENDENT PRACTITIONER for December, 1885. The editor has exercised his prerogative in omitting from Dr. Newkirk's manuscript some of the complimentary passages.

